# Breaking barriers: a statistical and machine learning-based hybrid system for predicting dementia

**DOI:** 10.3389/fbioe.2023.1336255

**Published:** 2024-01-08

**Authors:** Ashir Javeed, Peter Anderberg, Ahmad Nauman Ghazi, Adeeb Noor, Sölve Elmståhl, Johan Sanmartin Berglund

**Affiliations:** ^1^ Department of Health, Blekinge Institute of Technology, Karlskrona, Sweden; ^2^ School of Health Sciences, University of Skövde, Skövde, Sweden; ^3^ Department of Software Engineering, Blekinge Institute of Technology, Karlskrona, Sweden; ^4^ Department of Information Technology, Faculty of Computing and Information Technology, King Abdulaziz University, Jeddah, Saudi Arabia; ^5^ EpiHealth: Epidemiology for Health, Lund University, SUS Malmö, Malmö, Sweden

**Keywords:** dementia, voting classifier, F-score, machine learning, feature selection

## Abstract

**Introduction:** Dementia is a condition (a collection of related signs and symptoms) that causes a continuing deterioration in cognitive function, and millions of people are impacted by dementia every year as the world population continues to rise. Conventional approaches for determining dementia rely primarily on clinical examinations, analyzing medical records, and administering cognitive and neuropsychological testing. However, these methods are time-consuming and costly in terms of treatment. Therefore, this study aims to present a noninvasive method for the early prediction of dementia so that preventive steps should be taken to avoid dementia.

**Methods:** We developed a hybrid diagnostic system based on statistical and machine learning (ML) methods that used patient electronic health records to predict dementia. The dataset used for this study was obtained from the Swedish National Study on Aging and Care (SNAC), with a sample size of 43040 and 75 features. The newly constructed diagnostic extracts a subset of useful features from the dataset through a statistical method (F-score). For the classification, we developed an ensemble voting classifier based on five different ML models: decision tree (DT), naive Bayes (NB), logistic regression (LR), support vector machines (SVM), and random forest (RF). To address the problem of ML model overfitting, we used a cross-validation approach to evaluate the performance of the proposed diagnostic system. Various assessment measures, such as accuracy, sensitivity, specificity, receiver operating characteristic (ROC) curve, and Matthew’s correlation coefficient (MCC), were used to thoroughly validate the devised diagnostic system’s efficiency.

**Results:** According to the experimental results, the proposed diagnostic method achieved the best accuracy of 98.25%, as well as sensitivity of 97.44%, specificity of 95.744%, and MCC of 0.7535.

**Discussion:** The effectiveness of the proposed diagnostic approach is compared to various cutting-edge feature selection techniques and baseline ML models. From experimental results, it is evident that the proposed diagnostic system outperformed the prior feature selection strategies and baseline ML models regarding accuracy.

## 1 Introduction

Dementia is a severe neurological condition that causes memory, thinking, behavioral issues, and a steady decline in cognitive abilities (Creavin et al., 2016). Worldwide, millions of people are impacted by dementia. Around the globe today, 50 million people are thought to be affected by dementia. Dementia incidence is expected to triple by 2050. The prevalence of dementia will keep rising as the global population ages, putting enormous strain on healthcare systems worldwide (Iadecola, 2016). Early dementia diagnosis and prediction are crucial because they allow for quick intervention, improved patient treatment, and possible preventive measures; thus, prevention is essential for addressing this public health issue (Nichols et al., 2022).

The standard procedures for diagnosing dementia rely on clinical evaluations, which can be arbitrary and vulnerable to discrepancies between various assessors. These evaluations involve reviewing the medical records and conducting cognitive and neuropsychological tests Hsiu et al. (2022). Cognitive diagnostic tests or pathological characteristics diagnose dementia in its early stages. Pathological features can be found through neuroimaging. The alteration in neuronal structure is examined using magnetic resonance imaging (MRI) Studholme et al. (2004); Duchesne et al. (2008). These techniques are helpful but cannot address the minor alterations in brain activity that characterize dementia’s early stages. Electroencephalography (EEG) is another method to assess individuals in the initial phases of dementia Ahiskali et al. (2009). EEG and MRI imaging were coupled by Patel et al. to enhance the identification of dementia in its early stages Patel et al. (2008). However, due to the unacceptably high cost of testing and the excessively drawn-out and intrusive nature of the testing process, such instruments are insufficient for diagnosing dementia. Additionally, new studies advise using computed tomography (CT) or MRI of the brain to rule out structural explanations for the clinical phenotype. According to estimates, primary care physicians misdiagnose between 29% and 76% of people with dementia or are likely to develop dementia Patnode et al. (2020). This highlights the critical need for new diagnostic methods to identify dementia in its earliest stages reliably. Recent years have seen the emergence of machine learning (ML) as a potent tool for predictive analytics and pattern identification, providing exciting chances for advancements in this field Tanveer et al. (2020). Large-scale data sets can be examined by ML algorithms, which can also reveal hidden patterns that were previously undetected and make predictions. Numerous data sources, such as genetic markers, brain imaging data, lifestyle factors, and neuropsychological tests, have been used by researchers to test the efficacy of ML for dementia detection (Basheer et al., 2021). There are substantial potential advantages to ML’s capacity to identify dementia with early onset. Through prompt action when a disease is initially identified, it is possible to treat it optimally and enhance patients’ quality of life. Early dementia prediction aids researchers in their search for novel biomarkers and drug targets that will enable them to create more potent treatments (Spooner et al., 2020). There are still difficulties utilizing ML for dementia prediction despite the encouraging findings. For the efficient training and validation of ML models, big, meticulously assembled datasets covering a variety of variables are required (Sivakani and Ansari, 2020).

The aim and purpose of this study is given as follows:

1. Constructing a dataset for dementia by integrating data from four distinct SNAC sites (Blekinge, Kungsholmen, and Skåne) employing data integration and harmonization criteria.
2. Significant features are selected from the dataset using a statistical method (F-score).
3. For the classification of dementia, an ensemble voting classifier based on DT, NB, SVM, LR, and RF was constructed.
4. The effectiveness of the proposed hybrid system, which combines an ensemble voting classifier and a statistical method (F-score), is also evaluated in comparison to three other feature selection techniques.
5. Experimental results show that the proposed model outperforms the baseline machine learning models, such as AdaBoost, Random Forest, Support Vector Machines, Linear Regression, Logistic Regression, Naive Bayes, and Decision Tree, according to the three commonly used evaluation metrics of accuracy, ROC curves, and AUC.

### 1.1 Literature review

Numerous studies have been conducted on applying ML approaches to solve problems across various medical applications and disease prediction. Researchers have developed several ML and deep learning (DL) based algorithms for the early prediction of dementia, such as Salihović et al. (Salihović et al., 2018), discovered dementia predictors and deficits in multiple cognitive functions in vascular cognitive diseases. A recent study by Nyholm et al. (Nyholm et al., 2023) used machine learning to identify the risk factors for early prediction of dementia based on sleep disturbances in older adults. Wang et al. (Wang et al., 2019), examined the association between the difference in expected and chronological brain age and the development of dementia in a large population-based cohort of adult and older individuals using a deep learning model. According to the outcomes, the difference between expected and historical brain ages is a biomarker associated with dementia risk. It could be used as an additional biomarker for dementia threat assessment. Shigemizu (Shigemizu et al., 2019) constructed an optimal risk prediction model based on several ML methods, including penalized regression, RF, support vector machines, and gradient boosting decision tree, employing blood miRNA expression data from 478 Japanese adults. Ryu et al. (Ryu et al., 2020), address the subject of population aging and the growth of geriatric illnesses, notably dementia, which is lethal to the daily activities of the elderly. The authors provide a dementia prediction paradigm based on XGBoost, an ML algorithm that uses the derived variable extraction method. By extracting variable significance from traditional independent variables, they use gradient boosting to generate derived variables. The obtained variables’ findings are utilized to perform variable significance analysis, leading to the development of a Top-N group. Hyper-parameter alteration is used to achieve the best efficiency compatible with the data features for each Top-N group. The authors compare the performance of the proposed model to that of current ML classification methods. The effects of biomarker-based dementia risk estimation on quality of life (QoL) in mild cognitive impairment (MCI) patients and their immediate relatives have not been adequately examined.

Furthermore, Rostamzadeh (Rostamzadeh et al., 2021) provided empirical information on the effects of prediction on QoL and developed an ethical and legal framework for biomarker-based dementia risk assessment in MCI. Kühnel et al. (Kühnel et al., 2021), conducted a study to develop and validate a continuous biomarker-based model for estimating an individual’s cognitive level at any point in the future. Ghazal et al. (Ghazal et al., 2022), used ML classifiers to predict cancer, dementia, and diabetes using different datasets. Their proposed approach for multiclass classification used support vector machines (SVM) and K-nearest neighbor (KNN) ML algorithms to forecast three circumstances and compare the accuracy of these tactics.

For reliable dementia prediction, Javeed et al. (Javeed et al., 2022b), presented a hybrid diagnostic system based on ML algorithms. In their proposed method, they constructed an autoencoder that extracted features from the dataset, and an ensemble learning model was used for classification. In another study, Javeed et al. (Javeed et al., 2023d), presented a pair of automated diagnostic systems that use genetic algorithms for feature selection. At the same time, artificial neural networks (ANN) and deep neural networks (DNN) are used for dementia classification. Based on a genetic algorithm and a deep neural network, the suggested model had the highest accuracy of 93.36%, sensitivity of 93.15%, and specificity of 91.59%. Moreover, ML models tend to favor the majority class in the dataset. To solve this problem, Javeed et al. (Javeed et al., 2023e) proposed a diagnostic system for the early detection of dementia using an adaptive synthetic sampling technique (ADASYN) to solve the problem of imbalance in the dataset. They proposed novel feature extraction techniques, namely, feature extraction batteries (FEB) and optimized support vector machines (SVM) using radical basis functions (RBF), for dementia classification. The grid search method was used to calibrate the SVM hyperparameters. Their proposed model (FEB-SVM) increased the dementia prediction accuracy of the standard SVM by 6%. The proposed model (FEB-SVM) achieved a training accuracy of 98.28% and a test accuracy of 93.92%. The proposed approach achieved a precision of 91.80%, a recall of 86.59%, and an F1 score of 89.12%. M. A. Maito et al. (Maito et al., 2023) presented a fully automated computational approach based on classical statistical and machine learning methods for dementia prediction by identification of risk factors for dementia. The classification of Alzheimer’s disease (AD) and frontotemporal dementia (FTD) patients was shown to be accurate based on the results. With an accuracy of 0.91%, a machine learning model generated the optimal values to distinguish AD patients from FTD patients. M. Bucholc et al. (Bucholc et al., 2023) proposed a novel prognostic machine learning (ML) framework to identify mild cognitive impairment (MCI) patients who are susceptible to dementia by utilizing longitudinal data encoded in effective, affordable, and non-invasive markers. By their proposed method, RF and ensemble models had the highest reported accuracy, at 87.5% and 86.8%, respectively. Another study by Javeed et al. (Javeed et al., 2023c) aims to thoroughly evaluate the automated diagnostic systems previously presented by the researchers based on ML, using multiple data modalities such as images, medical variables, and audio data.

## 2 Materials and methods

### 2.1 Dataset description

The data for this study was obtained from the Swedish National Study on Ageing and Care (SNAC). In 1999, the Swedish Ministry of Social Affairs launched and funded a nationwide initiative to monitor and analyze the Swedish elderly care system. Four longitudinal, individual-based data-gathering projects characterizing the aging process and embracing the whole care system have been launched to accomplish these goals. The Swedish National Study on Aging and Care (SNAC) was the name given to this initiative. The SNAC is a long-running organization that collects multimodal data from Sweden’s aging population to offer reliable, efficient, and long-term data sets for aging research (Lagergren et al., 2004).

The SNAC program was developed to assess the quality of healthcare provided to older adults in multiple ways. Medical records, social variables, lifestyle factors, metacognitive data, and physical examination are only a few topics covered by SNAC’s databases. As a result, variables were chosen from the SNAC databases (Blekinge, Kungsholmen, and Skåne) based on previously published research in eight areas, including demographics, social factors, lifestyle, medical history, physical exam, biochemical testing, psychological exam, and evaluation of various health devices (Arvanitakis et al., 2019; Yu et al., 2020). In total, 75 variables were selected from the areas mentioned above. Table 1 provides the overview of selected variables from the SNAC databases.

**TABLE 1:** Overview of selected variables (features).

Variable_Category	Variable_Names	Sum
Demographic	Gender, Age	02
Social	Support Network, Education, Loneliness, Religious Belief, Social Network, Voluntary Association, Religious Activities	07
Lifestyle	Physical-Workload, Past Smoker, Physically Demanding Activities, Present Smoker, Alcohol Quantity, Leisure Activities, Number of Cigarettes a Day, Social Activities, Work Status, Alcohol Consumption, Light Exercise	11
Medical History	High Blood Pressure, Myocardial Infarction, Head Trauma, Arrhythmia, Sleep Apnea, TIA/RIND, Diabetes Type 1, Stroke, Thyroid Disease, Family History of Importance, Cancer, Epilepsy, Number of Medications, Diabetes Type 2, Parkinson’s Disease, Other Psychiatric Diseases, Snoring, Atrial Fibrillation, Hip Fracture, Cardiovascular Ischemia, Heart Failure, Developmental Disabilities, Depression	22
Physical Examination	Blood Pressure on the Right Arm, Number of Teeth, Body Mass Index (BMI), Assessment of Rising from a Chair, Heart Rate Sitting, Heart Rate Lying, Hand Strength in Right Arm in a 10s Interval, Hand Strength in Left Arm in a 10s Interval, Single-Leg Standing with Right Leg, Single Leg Standing with Left Leg, Pain in the last 4 weeks, Dental Prosthesis, Feeling of Safety from Rising from a Chair	13
Biochemical Test	C-Reactive Protein Analysis, Hemoglobin Analysis	02
Psychological	Sense of Identity, Memory Loss, Personality Change, Memory Decline, Abstract Thinking, Memory Decline 2	06
Health Instruments	Backwards Digit Span Test, Digit Span Test, Livingston Index, Sense of Coherence EQ5D Test, Instrumental Activities of Daily Living, Mini-Mental State Examination, Activities of Daily Living, Comprehensive Psychopathological Rating Scale, Physical Composite Score of the SF-12 Health Survey, Clock Drawing Test, Mental Composite Score of the SF-12 Health Survey	12

**FIGURE 1:**
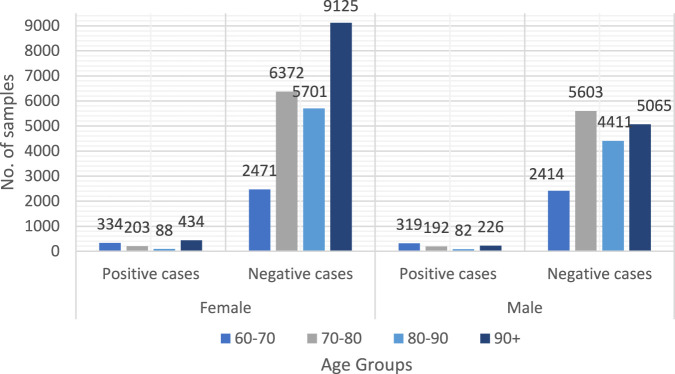
Samples overview in the collected dataset.

For this investigation, we acquired data from three SNAC facilities (Blekinge, Kungsholmen, and Skåne). Forty-three thousand forty data samples were collected, with 3,461 coming from SNAC-Kungsholmen, 7,304 from SNAC-Blekinge, and 32,275 from SNAC-Skåne. The primary purpose of the data collection was to integrate and harmonize the SNAC data from the three sites. The dataset was standardized by following the harmonization rule for the dataset’s variables. In the acquired dataset, there are 18,312 men and 24,728 women. Only 819 of the 18,312 males and 1,059 of the 24,728 females are affected by dementia. Table 2 displays the demographic information for the participant group. Because this study is based on broad criteria, no boundaries exist between urban and rural areas. Subjects excluded from this study based on the following criteria: Participants with dementia at baseline; participants with missing data for the outcome variable (dementia diagnosis); participants with more than 10% missing data for the entry variable; participants who died before the 10-year study or were diagnosed with advanced dementia were excluded (Figure 1) group.

**TABLE 2:** Summary of samples population.

	Female		Male	
Age_Group	+ve	-ve	+ve	-ve
60–70	334	2,471	319	2,414
70–80	203	6,372	192	5,603
80–90	88	5,701	82	4,411
90+	434	9,125	226	5,065
Total	1,059	23,669	819	17,493

### 2.2 Methodology

Real-world datasets come in a variety of sizes and forms. As a result, their nature imposes several significant limits on both learning models and feature selection techniques (Liu and Yu, 2005). Sample sizes and feature counts for datasets may be substantial, and problems with redundant, noisy, multivariate, and nonlinear scenarios may also arise. As a result, most currently used methods need help to solve these issues. Additionally, there is no such thing as “the best feature selection method” in general, which makes it challenging for users to choose one way over another. A user is expected to comprehend the technical specifics of the various algorithms and have a thorough understanding of each dataset’s domain and features to make the best decision (Tuv et al., 2009).

Many businesses today depend on machine learning techniques to extract meaningful data and expertise from escalating large datasets. For classification problems, feature selection methods are employed to mine the most relevant features in a feature space (Ali et al., 2019b; Akbar et al., 2020; Javeed et al., 2020; 2023d). In this sense, we employed a feature ranking method based on the statistical technique F-score. The F-score based on the feature ranking model (Chen and Lin, 2006) measures the discriminating between two sets of real numbers. If the number of samples associated with healthy participants is *v*_+_ and the number of samples of the patient group is *v*_−_ for a specific dataset with *l*_*i*_, i = 1, 2, 3,…, n occurrences, the F-score of the *n*th feature is determined as:

$$M_{1}=\frac{1}{\left(v_{+}-1\right)}\overset{v_{+}}{\underset{i=1}{\sum }}{\left({\bar{l}}_{i,n}^{\left(+\right)}-{\bar{l}}_{n}^{\left(+\right)}\right)}^{2}$$ (1)

$$M_{2}=\frac{1}{\left(v_{+}-1\right)}\overset{v_{-}}{\underset{i=1}{\sum }}{\left({\bar{l}}_{i,n}^{\left(-\right)}-{\bar{l}}_{n}^{\left(-\right)}\right)}^{2}$$ (2)

From Eqs 1 and 2, we get Eq. (3)

$$H\left(n\right)=\frac{{\left({\bar{l}}_{n}^{\left(+\right)}-{\bar{l}}_{n}\right)}^{2}+{\left({\bar{l}}_{n}^{\left(-\right)}-{\bar{l}}_{n}\right)}^{2}}{M_{1}+M_{2}}$$ (3)

Here, ${\bar{l}}_{n}$ , ${\bar{l}}_{n}^{+}$ and ${\bar{l}}_{n}^{-}$ are means of the *n* ^*th*^ feature of the positive and negative samples in the complete dataset. Furthermore, ${\bar{l}}_{n}^{-}$ is *n* ^*th*^ feature of the *i*th negative sample, and ${\bar{l}}_{n}^{+}$ is *i* ^*th*^ the positive sample of the *n* ^*th*^ feature. A further distinction between the positive and negative sets is made in 3’s numerator, while the denominator designates the singular value within each of the two sets (Akay, 2009). The F-score value is inversely correlated with a feature’s discriminative capacity. Following the statistical model-based ranking of features by their F-score, we must choose a threshold for the F-score, meaning only those features will be chosen if their F-score exceeds the threshold. In the current work, we use the hybrid grid search algorithm (HGSA) to find the ideal threshold to yield an ideal subset of the selected features. The extracted feature subset is supplied to the proposed ensemble model for classification.

**FIGURE 2:**
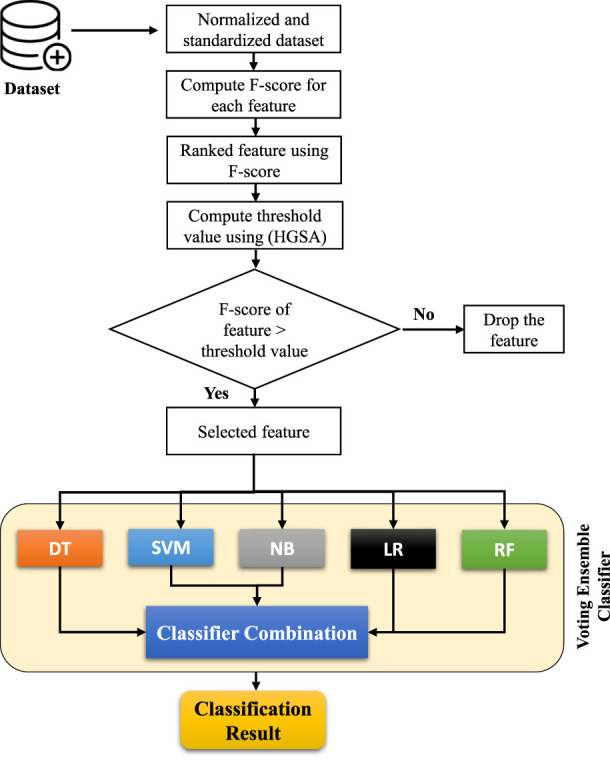
Working of proposed framework.

This study presents a framework using the F-score statistical method for feature selection and a voting ensemble classifier based on five machine learning (ML)-based models such as DT, SVM, NB, LR, and RF (Naseem et al., 2022) as shown in Figure 2. The complicated voting process determines the class with the most votes as the expected outcome (Bauer and Kohavi, 1999; Zhang et al., 2014). The proposed voting classifier used hard voting scheme for expected outcome. The next part also briefly explains each algorithm employed in our investigation.

### 2.3 Decision tree (DT)

DT comprises massive classifications of samples into specific categories Salzberg (1994). The utilization of specimens is made possible by patterns that combine nominal and numerical information to provide precise group descriptions. These indicators are then represented as models, producing decision frameworks or collections of if-then procedures that may be used to discriminate new samples, emphasizing the importance of creating clear and precise designs. The C4.5 calculus chooses the test that extracts the most data from a group of specimens without confining themselves to evaluating a single characteristic. They then apply equations based on theoretical data to estimate the ‘goodness’ of the test. Dealing with the overfitting and unknown values problem is DT’s main drawback. Unknown values are a problem that can be solved using the DT C4.5 approach, especially since samples with unknown values are usually ignored. A classifier that classifies every sample in the training set might not be as efficient as a DT. C4.5 implements an error rate-based pruning process for all subtrees to get around this. This method removes the subtree when the computed error is high. This method is more efficient and yields superior outcomes (Moslehi et al., 2022).

### 2.4 Support vector machines (SVM)

SVM is a supervised machine learning algorithm that constructs a linear discriminant function by using support vectors, which are an adequate number of samples. SVM resolved the linear constraints (Awad et al., 2015). A maximum hyperplane margin can be seen by partitioning the SVM data linearly into two classes. After choosing the suitable mapping, the new samples are linearly fitted or seem linearly separable in the high-level plane. The classification is done by graphing the best hyperplane, which can categorize the data objects according to their features (Javeed et al., 2023e). The following is a representation of the hyperplane:

ω.ρ+β=0 (4)

where the offset is represented by *β* and *ω* by the plane’s normal vector. Following the formation of the hyperplane, classifications based on the input vectors can be made. The following is a representation of the prediction hypothesis:

$$H\left(x_{i}\right)=\left\{\begin{array}{c} +1if\omega .x+\beta \geq 0\\ -1if\omega .x+\beta <0 \end{array}\right\}$$ (5)

When the input point is either above or on the hyperplane, it is categorized as positive, or *ω*.*x* + *β* ≥ 0. If it is under the hyperplane, it is defined as negative, or *ω*.*x* + *β* < 0. In the SVM, improvements are typically made by increasing the hyperplane’s separation from the support vectors.

### 2.5 Naive Bayes (NB)

The NB method is proposed using the Bayes theorem (Yager, 2006). The Bayes theorem and the precise processes can be used to revise the NB classifier in the following ways (Zheng et al., 2018). We conclude that a training set of examples *S* exists. These specimens bear group markings. The names of the groupings are *G*_1_, *G*_2_, … , *G*_*n*_. Every specimen is an agent with n dimensions, denoted by formula *D* = *d*_1_, *d*_2_, … , *d*_*n*_. It claims that because D has n dimensions, it has n characteristics. If the likelihood that group i depends on a given specimen, D, is higher than the likelihood that each of the other groups depends on D, then D is projected to belong to group *G*_*i*_, as given:

$$P\left(G_{i}|D\right)>P\left(G_{j}|D\right)for1\leq j\geq k_{j}=i$$ (6)

*P* (*G*_*i*_|*D*) is determined by the Bayes’ Theorem as follows:

$$P\left(G_{i}|D\right)=P\left(D|G_{j}\right)P\left(G_{i}/P\left(D\right)\right)$$ (7)

### 2.6 Logistic regression (LR)

LR is a supervised classification technique that predicts a category based on input attributes. LR is a predictive approach that makes predictions using probability values ranging from 0 to 1. As a result, an S-curve, also known as the sigmoid function, is formed. If the anticipated probability value exceeds a certain threshold, it is classified as positive; otherwise, it is classified as negative (Boateng and Abaye, 2019). The LR can be calculated using the formula below. A straight-line’s equation for LR is:

$$N=\alpha_{0}+\alpha_{1}\beta_{1}+\alpha_{2}\beta_{2}+\cdots +\alpha_{n}\beta_{n}$$ (8)

If the value of *N* is between 0 and 1, divide *N* by 1 − *N*.

$$\left[\frac{N}{1-N}\right]=\alpha_{0}+\alpha_{1}\beta_{1}+\alpha_{2}\beta_{2}+\cdots +\alpha_{n}$$ (9)

### 2.7 Random forest (RF)

From the feature vectors, the RF approach generates n-tree bootstrap samples. Each sample is used to build a classification tree that has not been trimmed. Each tree node evaluates a random collection of ‘F’ features and chooses the optimal split from them (Javeed et al., 2019; 2022a). This algorithm predicts the class of new, unknown data by aggregating the predictors of n trees using the majority voting technique (Paul et al., 2018).

Two hyperparameters are crucial for the classification job by RF model, such as D, the depth of each tree, and E, the number of trees making up the forest (Liu et al., 2015; Javeed et al., 2023f). In order to guarantee the enhanced performance of the random forest model, the best E and D were found in this study using the random search algorithm (RSA). In addition, a new sample is added, and an RF model is created. In the same manner, the decision tree determines and evaluates the new sample type. The final classification of a sample can be ascertained using the total number of votes cast in the decision tree within the forest. The bootstrap technique builds the RF formation trees from repeated samples by using training data. To apply the replacement method for model interactions, bootstrap is a straightforward and practical solution (Aprilliani and Rustam, 2018). Using bootstrap random sampling, a predetermined number of samples are taken from the training set. The number of samples that were extracted, the number of samples that were returned to the training set, and the number of bootstrap samples that were produced. It is also possible that the extracted samples will be re-sampled once the training set is returned. Thus, it is better to sample the previously extracted samples after storing them.

## 3 Experimental results

### 3.1 Evaluation metric

Several validation techniques, including holdout validation and cross-validation, are used in data mining and machine learning to assess how well a developed ML model performs. The cross-validation method has certain advantages over the holdout method, such as each partition of the data set used for training and testing of the ML models (Javeed et al., 2023b). Hence, to validate the performance of the proposed method for the prediction of dementia, we employed cross-validation schemes (Ali et al., 2019b; Liu et al., 2023b; Saleem et al., 2023).

The performance of the newly proposed method is assessed on several evaluation metrics, such as accuracy, sensitivity, specificity, and area under the curve (AUC), by employing the receiver operating characteristic curve (ROC) (Ali et al., 2019a; Liu et al., 2023a). Accuracy is given as follows:

$$Accuracy=\frac{No.\;of\;correct\;prediction}{Total\;samples}$$ (10)

Sensitivity and specificity are defined as follows:

$$Sensitivity=\frac{TP}{TP+FN}$$ (11)

$$Specificity=\frac{TN}{TN+FP}$$ (12)

When evaluating a classification model, TN represents true negatives, FN represents false negatives, FP represents false positives, and TP represents true positives.

The performance of predictive models must be measured using statistical analysis. In our statistical analysis of binary classification, we employed the Matthews correlation coefficient (MCC). The test’s accuracy is ascertained using MCC, a variable with values ranging from −1 to 1. Where −1 represents poorer predictions and 1 indicates exact predictions. The mathematical formulation of MCC is given as follows:

$$MCC=\frac{TP\times TN-FP\times FN}{\sqrt{\left(TP+FP\right)\left(TP+FN\right)\left(TN+FP\right)\left(TN+FN\right)}}$$ (13)

## 4 Results

### 4.1 Experiments no: 1 perfromance of baseline ML models

**FIGURE 3:**
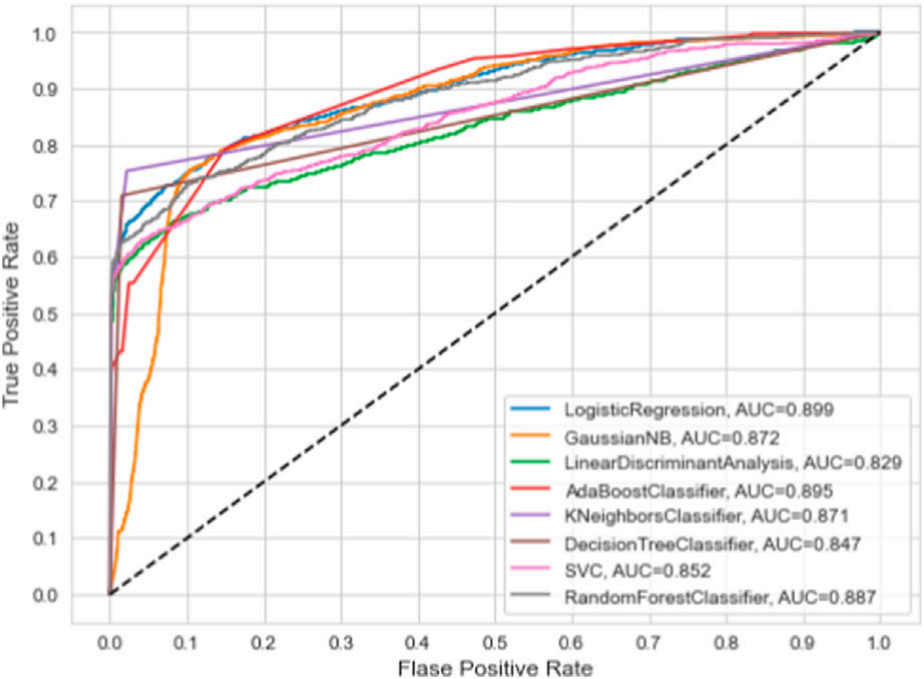
ROC curve analysis of baseline ML models.

This section summarizes the findings and presents the baseline models against which our suggested model was compared. We compare the proposed model’s performance to numerous machine learning classifiers and other approaches. All of the algorithms in this study were performed with their default parameters. Table 3 presents the results of baseline ML models on the given dataset. Furthermore, we have also employed the ROC curve to validate the performance results of based line ML models, as given in Figure 3. The ML model’s performance was evaluated by utilizing all the features present in the dataset. Table 3 and Figure 3 show that the highest accuracy and AUC are achieved through the LR model by using all the dataset features. While the worst performance for the prediction of dementia in terms of accuracy 82.90% is given by LDA.

**TABLE 3:** Performance of baseline ML Classifier.

ML models	Accuracy	Sensitivity	Specificity	MCC
LR	88.90	90.76	87.85	0.6699
NB	87.20	95.68	22.74	0.5423
LDA	82.90	88.14	72.49	0.6260
AdaBoost	89.50	92.51	84.52	0.6844
kNN	89.00	91.42	88.00	0.6733
DT	87.10	86.41	90.57	0.6577
SVM	85.20	88.00	82.36	0.6468
RF	88.70	92.87	84.14	0.6600

In the next phase, we validate the performance of the proposed method, where useful features are selected from the dataset by employing the statistical method F-score. We constructed a voting classifier based on DT, SVM, NB, LR, and RF for the classification job. We have extracted different sizes of subsets of features from the dataset and measured their performance using a voting classifier. Table 4 presents the performance of the proposed model on different subsets of features. The performance of the proposed model is measured in terms of accuracy, sensitivity, specificity, and MCC.

**TABLE 4:** Performance of proposed model.

Sub_Fe.	Accuracy	Sensitivity	Specificity	MCC
02	94.64	95.64	48.50	0.6513
04	92.10	96.42	79.00	0.5944
06	95.62	96.82	92.50	0.6852
07	95.05	96.91	85.45	0.6600
08	98.12	98.00	93.00	0.7515
09	98.25	97.44	95.74	0.7535
10	97.90	96.77	94.40	0.7412
12	98.00	97.69	95.00	0.7500

### 4.2 Experiments no: 2 perfromance of propsoed model

In this section, we evaluate the performance of the proposed model, where features from the dataset are selected based on the statistical method (F-score) and the ensemble voting classifier performs the classification task. We have stacked five different ML models for the voting classifier, i.e., DT, SVM, NB, LR, and RF. To validate the efficiency of the newly developed method for the prediction of dementia, we employed a cross-validation scheme (k = 5) to avoid the problem of model overfitting. Table 4 presents the performance of the proposed model based on accuracy, sensitivity, specificity, and MCC by using a subset of features (Sub_Fe) extracted by the F-score. Table 4 shows that the highest accuracy achieved by the proposed model was 98.25%, using only nine features from the dataset.

**FIGURE 4:**
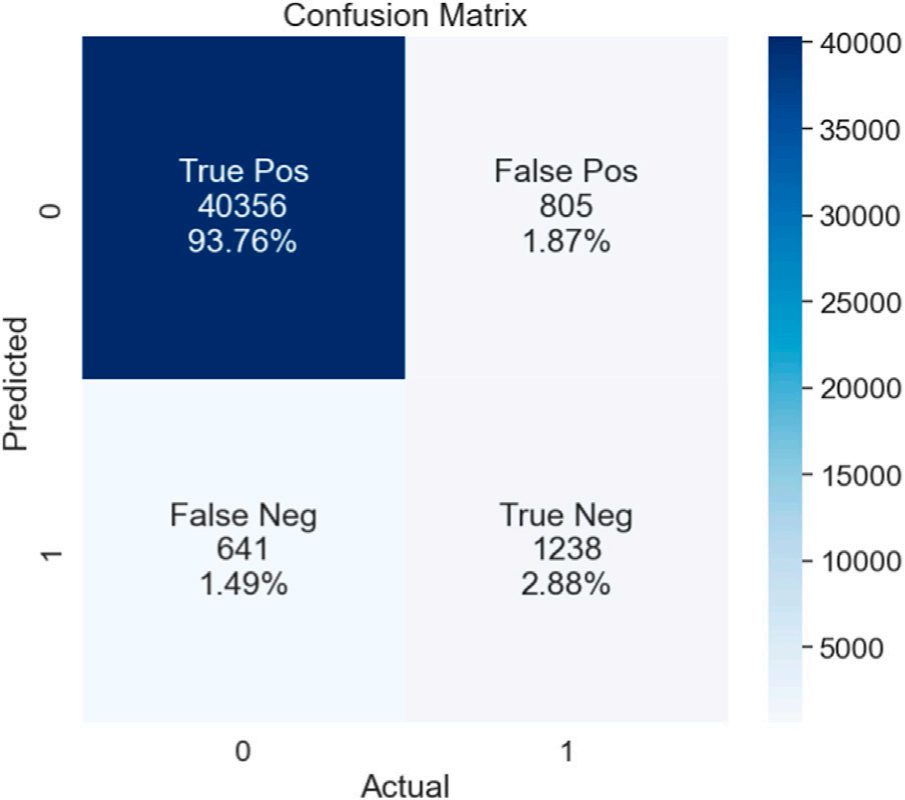
Confusion matrix.

**FIGURE 5:**
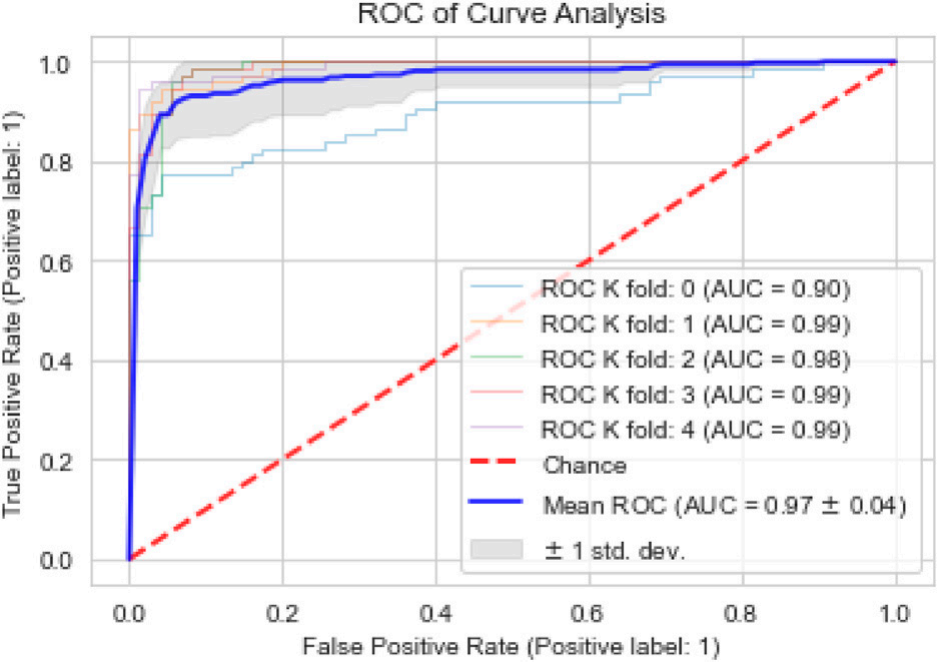
ROC curve analysis.

The performance of the proposed model is also assessed based on a confusion matrix, as seen in Figure 4. In binary classification problems, the efficiency of ML models is often validated using the ROC curve. A larger area under the curve (AUC) indicates a more efficient model. We also used the ROC curve to evaluate our proposed model’s performance. Figure 5 shows that our model achieved the highest AUC of 97% through the cross-validation (k = 5) scheme.

### 4.3 Experiments no: 3 performance of other feature selection methods

In this section, we conducted the experiment where the performance of different feature selection methods, i.e., chi-square test, mutual information (mutinfo), recursive feature elimination (RFE), least absolute shrinkage, and selection operator (lasso), was evaluated with a constructed voting classifier for the classification.

**FIGURE 6:**
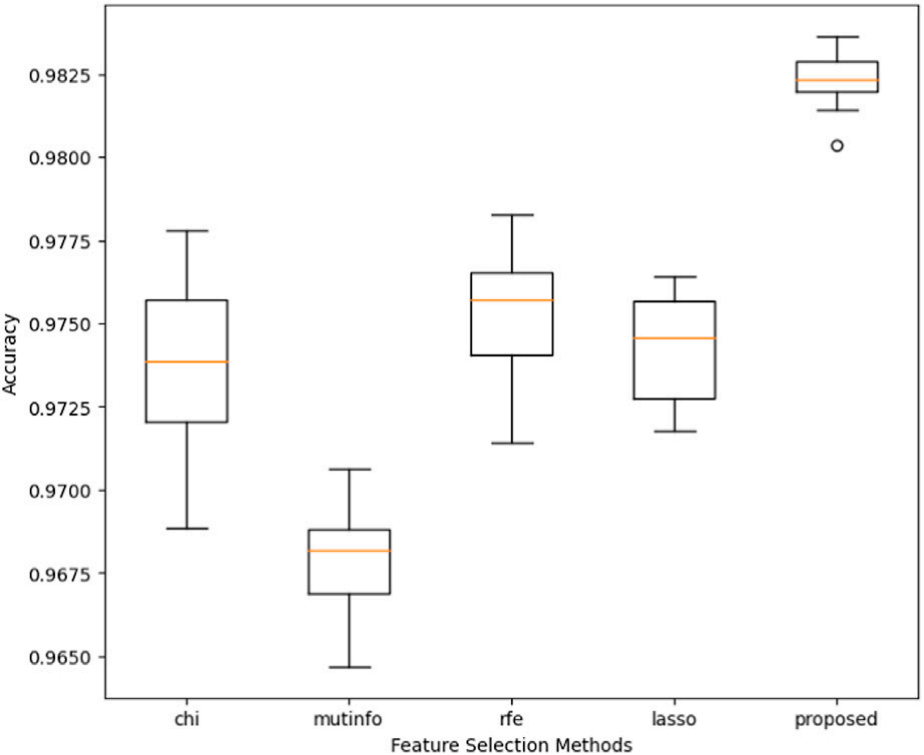
Performance comparison of the proposed method with other feature selection methods.

Furthermore, we compared the performance of the proposed model with the feature mentioned above selection algorithms. Figure 6 presents the performance comparison regarding the proposed model’s accuracy and the four state-of-the-art feature selection techniques (chi-square, mutinfo, RFE, and lasso). The accuracy is measured through the cross-validation scheme, where the value of k was set to 10. The proposed model achieved the highest accuracy of 98.25%, while mutinfo, along with the constructed voting classifier, obtained the lowest accuracy of 96.75%.

## 5 Discussion

For this work, we developed a large dataset from the Swedish National Study on Ageing and Care (SNAC) for the early prediction of dementia and its risk factors. SNAC is a cohort-based study collecting data from older Swedish adults since 2002. For this study, we gathered data from three distinct locations in Sweden (Blekinge, Kungsholmen, and Skåne). In total, 43,040 data samples were collected, comprising 75 features for each sample. The description of selected features for this study is given in Table 2, where features belong to eight different categories such as lifestyle (11), demography (02), social (07), medical history (22), health instruments (12), biochemical tests (02), psychological (06) and physical examination (13). We employed data harmonization rules to integrate the data from three SNAC centers. After data collection, we clean the dataset by performing data standardization and normalization techniques. This study aimed to design a diagnostic system that can predict the early onset of dementia in older adults and detect the risk factors that cause dementia. For this purpose, we proposed a hybrid diagnostic system based on statistical methods and machine learning techniques. Form feature space, highly significant features are selected through statistical method (F-score). We designed an ensemble voting classifier based on 5 ML classifiers (DT, NB, SVM, LR, and RF) for the classification task. The proposed system generated a subset of significant features, which were tested by constructed voting classifiers to accurately predict dementia. As dementia is rare, the number of instances of dementia in comparison to the healthy instances is less. The ML models tend to overfit due to the majority class in the dataset.

To avoid this problem, we deployed several evaluation metrics based on a cross-validation scheme to assess the efficiency of the newly constructed model. From Table 4, it is evident that the proposed model achieved the highest accuracy of 98.25% by using only nine features from the dataset. Table 5 describes nine highly significant features selected by the proposed model that help to predict dementia. Most of the features selected by the proposed model for the prediction of dementia are related to subject health conditions such as heart disease, TIA/RIND, head trauma, psychosis, and arrhythmia. While few are associated with social interaction, such as receiving low assistance regarding personal care and entertaining with abstract ideas, One selected feature (the mental rotation test) is related to psychology, where subjects with low scores are more prone to dementia. Subjects with a massive BMI also have dementia in older age.

**TABLE 5:** Significant features selected by the proposed model.

Feature.	Description
E18	Heart disease
E19	TIA/RIND*
E23	Head Trauma with loss of consciousness/syncope
E44	Psychosis
E58_C	ECG - Arrhythmia
F155	I happily, and often, entertain myself with abstract ideas or theories
FP10	Mental Rotations Test- Total number of correct respones (0–10)
H27	Receive not enough assistance regarding personal care
BMI	Body Mass Index

*TIA/RIND: transient ischaemic attack reversible ischaemic neurological deficit.

The constructed voting classifier also evaluates the performance of other feature selection (chi square, ref, mutinfo, lasso) methods. Figure 6 shows that the proposed model obtained the highest accuracy compared to the rest of the feature selection methods. Furthermore, we also evaluated the performance of the conventional ML models using all features from the dataset. Table 3 shows that the AdaBoost obtained the highest accuracy of 89.59% while LDA achieved the lowest accuracy of 82.90%.

Furthermore, we have also compared the performance of the proposed method’s classification accuracy to other methods in the literature using the dementia dataset. Table 6 provides a succinct summary of these approaches. The newly proposed method outperforms the eighteen recently proposed methods in terms of accurate prediction of dementia. Furthermore, the presented framework has two components, and they work in sequential coordination. The first component is used for the feature selection from the dataset, and the second component is employed for the classification. Thus, the computational complexity of the proposed framework is O(logn) .

**TABLE 6:** The proposed method’s classification accuracy compared to other methods in the literature using the EHR of dementia.

S.No	Study	Method	Validation	Accuracy (%)
01	Cho and Chen (2012)	PNNs	Holdout	83.00
02	Gurevich et al. (2017)	SVM	K-Fold	89.00
03	Stamate et al. (2018)	Gradient Boosting	K-Fold	88.00
04	Visser et al. (2019)	XGBoost + RF	K-Fold	88.00
05	Dallora et al. (2020)	DT	K-Fold	74.50
06	Karaglani et al. (2020)	RF	Holdout	84.60
07	Ryzhikova et al. (2021)	ANN + SVM	K-Fold	84.00
08	Salem et al. (2021)	RF	K-Fold	88.00
09	Garcia-Gutierrez et al. (2022)	GA	K-Fold	84.00
10	Hsiu et al. (2022)	MLP	K-Fold	70.32
11	Shahzad et al. (2022)	SVM	K-Fold	71.67
12	Javeed et al. (2022b)	Autoencoder + Adaboost	Holdout	90.23
13	Bucholc et al. (2023)	Ensemble Model	K-Fold	87.50
14	Maito et al. (2023)	Anova test + SVM	K-Fold	91.00
15	Nyholm et al. (2023)	Gradient Boosting	K-Fold	92.90
16	Javeed et al. (2023d)	GA + DNN	Holdout	93.36
17	Javeed et al. (2023e)	FEB + SVM	Holdout	93.92
18	Javeed et al. (2023a)	LDA + DT	Holdout	97.77
19	Proposed Method (2023)	F-Score + Voting Classifier	K-Fold	98.25

Although the newly proposed method has shown evident performance in terms of accuracy, there are a few concerns that need to be addressed in future research work. One of the limitations of this study is that it uses only electronic health record data. Therefore, in the future, researchers should focus on multimodality datasets for the prediction of dementia. Hybrid diagnostic systems are complex in nature, especially in terms of computational and time complexity; thus, novel diagnostics should be developed in the future based on machine learning and deep learning that are simple and easily integrated into the real-world scenario.

## 6 Conclusion

This study presented a hybrid diagnostic system that helps predict dementia in its early stages by using the medical health records of older adults. The newly developed diagnostic system consists of two modules: statistical models and ensemble ML models. There are 75 features in the dataset. To eliminate the irrelevant features from the feature space, we deployed a statistical (F-score) model that helps construct a subset of useful features from the dataset. We developed a voting classifier based on DT, SVM, NB, LR, and RF for the classification. The extracted subset of features from the first module of the developed diagnostic system is fed into the second module for the classification of dementia. To assess the performance of the developed diagnostic system, we employed a cross-validation scheme to overcome the problem of ML model overfitting. Various evaluation metrics were adopted to rigorously validate the efficiency of the developed diagnostic system, such as accuracy, sensitivity, specificity, ROC, and MCC. The experimental results show that the proposed diagnostic system achieved an accuracy of 98.25%, with a sensitivity of 97.44%, a specificity of 95.74%, and an MCC of 0.7535. Furthermore, the performance of the proposed diagnostic system is also compared with the baseline ML models as well as other state-of-the-art feature selection methods. In this context, four different feature selection methods, such as chi square, mutinfo, ref, and lasso, were tested along with the constructed voting ensemble classifier for the classification. Regarding accuracy, the proposed model outperformed the rest of the feature selection methods and baseline ML models.

## Data Availability

The raw data supporting the conclusion of this article will be made available by the authors, without undue reservation.
